# Prevalence of preterm, low birthweight, and small for gestational age delivery after breast cancer diagnosis: a population-based study

**DOI:** 10.1186/s13058-017-0803-z

**Published:** 2017-01-31

**Authors:** Kristin Zeneé Black, Hazel B. Nichols, Eugenia Eng, Diane Louise Rowley

**Affiliations:** 1 Department of Health Behavior, Gillings School of Global Public Health, University of North Carolina at Chapel Hill, 135 Dauer Drive, Campus Box 7440, Chapel Hill, NC 27599-7440 USA; 2 Department of Epidemiology, Gillings School of Global Public Health, University of North Carolina at Chapel Hill, 135 Dauer Drive, Campus Box 7435, Chapel Hill, NC 27599-7435 USA; 3 Department of Maternal and Child Health, Gillings School of Global Public Health, University of North Carolina at Chapel Hill, 135 Dauer Drive, Campus Box 7445, Chapel Hill, NC 27599-7445 USA

**Keywords:** Breast cancer, Premenopausal, Preterm birth, Low birthweight, Small for gestational age, Racial disparities

## Abstract

**Background:**

Black-white disparities in breast cancer incidence rates and birth outcomes raise concerns about potential disparities in the reproductive health of premenopausal breast cancer survivors. We examined the prevalence of preterm birth (PTB), low birthweight (LBW), and small for gestational age (SGA) by breast cancer history and effect modification by race.

**Methods:**

We analyzed linked North Carolina birth records and Central Cancer Registry files from 1990 to 2009 (*n* = 2,325,229). We used multivariable negative log-binomial regression to calculate prevalence ratios (PRs) and 95% confidence intervals (CIs) for the association between breast cancer history and PTB, LBW, and SGA.

**Results:**

Of 1,912,269 eligible births, 512 births were to mothers with a previous breast cancer diagnosis history. Average age at breast cancer diagnosis was 31.8 years (SD = 4.7). Mean time from diagnosis to delivery was 3.3 years (SD = 2.8). After multivariable adjustment, the PR was 1.67 (95% CI, 1.42–1.97) for PTB, 1.50 (95% CI, 1.23–1.84) for LBW, and 1.30 (95% CI, 1.05–1.61) for SGA comparing women with a breast cancer history to the general population. Among black mothers, the PRs associated with breast cancer history for PTB, LBW, and SGA were 1.31 (95% CI, 1.00–1.72), 1.49 (95% CI, 1.14–1.94), and 1.44 (95% CI, 1.11–1.87), respectively. The corresponding PRs among white mothers were 2.06 (95% CI, 1.67–2.54), 1.53 (95% CI, 1.12–2.08), and 1.10 (95% CI, 0.77–1.58), respectively. The interaction between breast cancer history and race was statistically significant for associations with PTB, but not for LBW or SGA.

**Conclusions:**

In our data, women with a breast cancer history were at higher risk of delivering a PTB, LBW, or SGA infant, especially if they received chemotherapy or gave birth within 2 years of their breast cancer diagnosis date.

## Background

With an increasing number of women delaying childbearing [1, 2] and improved breast cancer survival among women diagnosed before the age of 50 years [3], many young breast cancer patients face decisions at the time of diagnosis that may influence their future reproductive health. Beyond the risk of infertility [4–7], other adverse birth outcomes including preterm birth (PTB), low birthweight (LBW), and small for gestational age (SGA) may be more common among women with a prior breast cancer history [8]. The potential risk of adverse birth outcomes among breast cancer survivors has not been studied in the context of existing racial disparities in breast cancer incidence and birth outcomes in the USA.

Black women are more likely than their white counterparts to be diagnosed with breast cancer during their reproductive years [9] and deliver PTB, LBW, or SGA infants. Breast cancer diagnosed in young women, especially young black women, is more likely to be more aggressive and have a poorer prognosis than breast cancer diagnosed in older women and young white women [10–12]. In the general USA population, 9.6% of infants are delivered preterm (PTB; <37 weeks gestation) and 8.1% have a low birthweight (LBW; <2500 g) [2]. Rates of PTB, LBW, and SGA (i.e., sex-specific birthweight below the tenth percentile for gestational age) increase with maternal age and vary by race/ethnicity. For example, mothers aged 45 years or older compared to those aged 25–34 years have a higher portion of PTB (24.2 vs. 9.1%) and LBW (20.4 vs. 7.4%) deliveries [13]. Compared to white women, black women are 41% more likely to deliver a PTB infant, 63% more likely to deliver a LBW infant [2], and two to three times more likely to deliver a SGA infant [14, 15].

Breast cancer diagnosis and its treatment may have long-lasting effects on reproductive health outcomes. Alkylating agent-based chemotherapies can cause ovarian toxicity [5], including loss of mature ovarian follicles. During chemotherapy treatment, premenopausal women may experience temporary chemotherapy-related amenorrhea or premature ovarian failure [4–7]. After completion of chemotherapy, a 5- to 10-year course of adjuvant endocrine therapy is recommended for hormonally responsive tumors to improve relapse-free and overall survival [6, 7, 16]. Pregnancy is not recommended during the course of adjuvant endocrine therapy; therefore, some women may choose to not initiate or to interrupt endocrine therapy to achieve pregnancy [17].

Black-white disparities in breast cancer incidence rates and birth outcomes raise concerns about potential disparities in the reproductive health of premenopausal breast cancer survivors. Most previous studies have focused on survival rates among breast cancer survivors with a post-diagnosis pregnancy compared to those without [18–20]. Survival does not appear to be adversely affected by post-diagnosis pregnancy; however, this may be partially attributable to a “healthy mother effect” [18, 21, 22]. Few published studies have examined infant outcomes after a breast cancer diagnosis [8, 18, 20, 23–25]. Our study examined the prevalence of PTB, LBW, and SGA according to breast cancer history (i.e., a personal breast cancer diagnosis) prior to infant delivery in a population-based study in North Carolina (NC), and evaluated potential effect modification by race.

## Methods

This study was reviewed and approved by the University of North Carolina at Chapel Hill’s IRB and Office of Human Research Ethics (#14-1394).

### North Carolina Central Cancer Registry

The primary exposure for this analysis was breast cancer history prior to infant delivery. We identified breast cancer diagnoses during the study period (1990–2009) within the state-mandated North Carolina Central Cancer Registry (NC-CCR) [26]. The NC-CCR is a gold-certified North American Association of Central Cancer Registries (NAACCR) cancer registry within the Centers for Disease Control and Prevention’s National Program of Cancer Registries. We used topography site code C50 from the International Classification of Disease-Oncology third Edition [27] to identify first primary breast cancer diagnoses at ages 18–45 years. Additional NC-CCR variables included date of diagnosis, age at diagnosis, and receipt of treatment (e.g., surgery, chemotherapy, radiation, endocrine therapy).

### North Carolina birth certificate files

The study population for this analysis was comprised of all live, singleton births to NC residents during 1990–2009 (*n* = 2,325,229). Within 10 days of a delivery, a hospital administrator or person attending a non-hospital delivery (e.g., midwife) must file a birth certificate to the Department of Health and Human Services. The NC State Center for Health Statistics houses and manages these vital statistics records [28]. Further eligibility criteria included maternal ages 18–50 years (*n* = 2,213,487), and maternal ethnicity and race designated as non-Hispanic Black or White (*n* = 1,916,998). Latinas and other races/ethnicities were excluded because there was not sufficient power to examine these racial/ethnic categories independently. Women who experienced a stillbirth, miscarriage, or other adverse pregnancy outcome that did not result in a live birth are not included because they are not systematically captured in vital records data. We also excluded mothers who delivered infants at less than 20 weeks gestation or weighing less than 500 g because they were outside of the age and weight of viability. After these exclusions, 1,912,269 births contributed to the LBW analysis, 1,910,014 births contributed to the PTB analysis (2,255 births were missing gestational age), and 1,909,748 births contributed to the SGA analysis (2,521 births were missing gestational age or sex).

Information abstracted from the birth certificate files included the number of weeks of gestation at time of delivery, the infant’s weight in pounds and ounces, the maternal number of years of education, marital status at time of delivery, number of living children, race, ethnicity, number of cigarettes smoked per day during pregnancy, maternal age at delivery, and date of infant delivery.

### North Carolina Central Cancer Registry and vital records linkage

Birth certificate files were linked with the NC-CCR for the period of 1 January 1990 to 31 December 2009 by the NC State Center for Health Statistics. The linkage protocol applied a probabilistic algorithm using names and social security numbers in Link Plus (Centers for Disease Control and Prevention; Atlanta, GA, USA). Identifying information was redacted from the final dataset. Breast cancer history prior to infant delivery was defined as a maternal breast cancer diagnosis recorded in the NC-CCR that preceded the date of delivery in the birth certificate files. Two births by mothers with a breast cancer history were excluded because the diagnosis date was not available and the diagnosis age was above 45 years.

### Covariates

The risk of delivering a PTB, LBW, or SGA infant is known to vary by several maternal characteristics, including the age of the mother at the time of pregnancy and their race/ethnicity and socioeconomic status (SES) [14, 15, 29–34]. Studies that have focused on disparities in medical treatment and health outcomes have demonstrated that racial/ethnic and SES disparities are particularly apparent among cancer patients [35–39]. Therefore, maternal characteristics, race/ethnicity, and SES were important covariates to include in the analytical models of this study. Each covariate included in the analytical models is described below.

Maternal years of education were categorized as less than high school (≤11 years), high school diploma (12 years), at least some or graduated college (13–16 years), and professional/graduate degree (≥17 years). The marital status of the mother was abstracted from the birth certificate (married or not married). The number of living children was used to determine parity status as primiparous (i.e., no infant delivery prior to the current birth) or multiparous (i.e., a previous delivery in addition to the current birth). Maternal race/ethnicity is composited using the race and Hispanic ethnicity variables on the birth certificate. The number of cigarettes smoked per day during pregnancy was dichotomized as smoked during pregnancy (yes/no). The receipt of chemotherapy is derived from the first listed date of chemotherapy treatment or otherwise categorized as no chemotherapy. The length of time between diagnosis and infant delivery was calculated using the breast cancer diagnosis date of the mother and the date of infant delivery, and categorized as <2, 2–4.9, and ≥5 years. Maternal age at infant delivery (in years) was included in the analysis as a continuous variable.

### Outcomes

Preterm birth was defined as gestational age 20 to <37 weeks at delivery. Infant weight was calculated by converting weight in pounds and ounces to grams. Low birthweight was defined as <2500 g. Small for gestational age was defined as sex-specific birthweight below the tenth percentile for a given gestational age. Based on the continuous measure of birthweight for gestational age of Oken et al. [40], infant sex, gestational age, and weight in grams were used to calculate SGA.

### Statistical analysis

Multivariable negative log-binomial regression was used to calculate prevalence ratios (PRs) and 95% confidence intervals (CIs) for the association between breast cancer history and birth outcome (i.e., PTB, LBW, and SGA). We assessed and identified additional covariates as an effect measure modifier using likelihood ratio tests (e.g., race/ethnicity) with the a priori significance criteria set at 5% or as a confounder using 5% change in estimate tests (e.g., maternal age at infant delivery, education, marital status, parity, and smoking) for inclusion in multivariable models. Confounders that met the criteria for PTB, LBW, or SGA were adjusted for in analyses for all outcomes. We assessed statistical interaction between breast cancer history and race on the multiplicative (stratified) scales using cross-product interaction terms [41]. All analyses were performed using SAS statistical software version 9.4 (Cary, NC, USA).

## Results

Of the 1,912,269 eligible live births in NC during 1990–2009, 512 births were linked to mothers with a breast cancer history. The mean age at breast cancer diagnosis was 31.8 years (standard deviation (SD) = 4.7) and the average time from diagnosis to delivery was 3.3 years (SD = 2.8). Nearly half (49.4%) of mothers with a breast cancer history had a record of starting chemotherapy (Table 1).

**Table 1:** Characteristics of live births according to maternal breast cancer history at time of delivery, 1990–2009

	Breast cancer history (*n* = 512)		General population (*n* = 1,911,757)	
	*n*	%	*n*	%
Preterm birth				
Term (≥37 weeks gestation)	404	78.9	1,703,476	89.1
Preterm (<37 weeks gestation)	108	21.1	206,026	10.8
Missing	0	0.0	2255	0.1
Low birthweight				
Healthy weight (≥2500 g)	436	85.2	1,743,325	91.2
Low birthweight (<2500 g)	76	14.8	168,432	8.8
Missing	0	0.0	0	0.0
Small for gestational age				
Not small for gestational age	444	86.7	1,696,048	88.7
Small for gestational age	68	13.3	213,188	11.2
Missing	0	0.0	2521	0.1
Education				
Less than high school (≤11 years)	19	3.7	266,180	13.9
High school diploma (12 years)	123	24.0	662,969	34.7
Some or graduated college (13–16 years)	282	55.1	814,843	42.6
Professional/graduate degree (≥17 years)	87	17.0	164,664	8.6
Missing	1	0.2	3101	0.2
Marital status				
Married	404	78.9	1,320,768	69.1
Not married	108	21.1	590,559	30.9
Missing	0	0.0	430	0.02
Parity				
Primiparous (1 birth)	153	29.9	787,261	41.2
Multiparous (≥2 births)	359	70.1	1,124,496	58.8
Missing	0	0.0	0	0.0
Race/ethnicity				
White, non-Hispanic	324	63.3	1,382,978	72.3
Black, non-Hispanic	188	36.7	528,779	27.7
Missing	0	0.0	0	0.0
Smoking				
Non-smoker	463	90.4	1,605,661	84.0
Smoker	46	9.0	296,037	15.5
Missing	3	0.6	10,059	0.5
Maternal age at infant delivery (in years)				
Mean (standard deviation)	34.6 (4.7)		27.1 (5.7)	
Range	20.0–48.0		18.0–50.0	
Age at breast cancer diagnosis (in years)				
Mean (standard deviation)	31.8 (4.7)			
Range	18.7–44.3			
Chemotherapy				
Did not receive chemotherapy	259	50.6		
Received chemotherapy	253	49.4		
Missing	0	0.0		
Length of time between diagnosis and infant delivery				
<2 years	195	38.1		
2–4.9 years	197	38.5		
≥5 years	120	23.4		
Missing	0	0.0		

Overall, 10.8% of infants were PTB, 8.8% were LBW, and 11.2% were SGA. Among all births, 72.3% were to white women and 27.7% to black women. White women had a higher prevalence of PTB (6.8% vs. 3.9%), LBW (5.1% vs. 3.7%), and SGA (6.4% vs. 4.7%) compared to black women (data not shown). About 44.7% of LBW deliveries to women with a breast cancer history and 46.9% to women without a breast cancer history were SGA (data not shown). Compared to the general population of reproductive age mothers, mothers with a breast cancer history were older at the time of infant delivery, and more likely to have attended college, be married, and not smoke during pregnancy (Table 1).

We observed an increased risk of PTB, LBW, and SGA for births to women with a breast cancer history, especially for women who received chemotherapy treatment or gave birth within 2 years of their diagnosis date. After multivariable adjustment for maternal age, education, marital status, parity, race, and smoking, the PR associated with breast cancer history was 1.67 (95% CI, 1.42–1.97) for PTB, 1.50 (95% CI, 1.23–1.84) for LBW, and 1.30 (95% CI, 1.05–1.61) for SGA. The PR of PTB among births to mothers with a breast cancer history that received chemotherapy treatment was 2.17 (95% CI, 1.79–2.63) compared to the general population. The corresponding PRs were 1.92 (95% CI, 1.50–2.45) for LBW and 1.63 (95% CI, 1.25–2.13) for SGA. The PR of PTB among births that occurred within 2 years of the mother’s diagnosis date was 2.58 (95% CI, 2.12–3.15) compared to the general population. The corresponding PRs were 2.16 (95% CI, 1.64–2.85) for LBW and 1.36 (95% CI, 0.96–1.92) for SGA (Table 2).

**Table 2:** Age adjusted and multivariable PR and 95% CI for preterm birth, low birthweight, and small for gestational age

	Preterm birth (<37 weeks)			Low birthweight (<2500 g)			Small for gestational age (<10th percentile^c^)		
	*n*	Age adjusted^a^ PR (95% CI)	Multivariable^b^ PR (95% CI)	*n*	Age adjusted^a^ PR (95% CI)	Multivariable^b^ PR (95% CI)	*n*	Age adjusted^a^ PR (95% CI)	Multivariable^b^ PR (95% CI)
Breast cancer history									
No^d^	206,026	1.00	1.00	168,432	1.00	1.00	213,188	1.00	1.00
Yes	108	1.89 (1.60–2.24)	1.67 (1.42–1.97)	76	1.76 (1.43–2.16)	1.50 (1.23–1.84)	68	1.48 (1.19–1.85)	1.30 (1.05–1.61)
Chemotherapy									
No	38	1.31 (0.98–1.76)	1.17 (0.87–1.56)	28	1.28 (0.90–1.82)	1.10 (0.78–1.55)	26	1.14 (0.79–1.64)	0.98 (0.68–1.40)
Yes	70	2.48 (2.03–3.03)	2.17 (1.79–2.63)	48	2.24 (1.73–2.89)	1.92 (1.50–2.45)	42	1.83 (1.39–2.40)	1.63 (1.25–2.13)
Time between diagnosis and delivery									
<2 years	62	2.86 (2.33–3.52)	2.58 (2.12–3.15)	38	2.29 (1.72–3.04)	2.16 (1.64–2.85)	26	1.43 (1.00–2.04)	1.36 (0.96–1.92)
2–4.9 years	30	1.36 (0.98–1.90)	1.20 (0.86–1.66)	20	1.20 (0.79–1.82)	0.98 (0.65–1.47)	21	1.20 (0.80–1.79)	0.97 (0.66–1.44)
≥5 years	16	1.19 (0.75–1.87)	1.04 (0.66–1.63)	18	1.79 (1.17–2.74)	1.49 (0.98–2.26)	21	2.08 (1.41–3.06)	1.85 (1.27–2.71)

^a^Adjusted for mother’s age at infant delivery

^b^Adjusted for mother’s age at infant delivery, education, marital status, parity, race/ethnicity, and smoking

^c^Birthweight of infant is below the tenth percentile for the infant’s gestational age and sex

^d^Women with no breast cancer history serve as the reference group for all comparisons

*CI* confidence interval, *PR* prevalence ratio

In analyses stratified according to maternal race, PRs for the association between breast cancer history and PTB, LBW, and SGA among black mothers were 1.31 (95% CI, 1.00–1.72), 1.49 (95% CI, 1.14–1.94), and 1.44 (95% CI, 1.11–1.87), respectively. The corresponding PRs among white mothers were 2.06 (95% CI, 1.67–2.54), 1.53 (95% CI, 1.12–2.08), and 1.10 (95% CI, 0.77–1.58), respectively. The interaction between breast cancer history and race was statistically significant for associations with PTB (*P* interaction = 0.01), but not for LBW (*P* interaction = 0.9) or SGA (*P* interaction = 0.2).

Compared to white mothers in the general population, the PRs for PTB were 1.45 (95% CI, 1.44–1.47) for black mothers without a breast cancer history and 1.90 (95% CI, 1.45–2.50) for black mothers with a breast cancer history. The PRs for LBW were 1.82 (95% CI, 1.80–1.84) for black mothers without a breast cancer history, and 2.71 (95% CI, 2.08–3.54) for black mothers with a breast cancer history. The PRs for SGA were 1.82 (95% CI, 1.80–1.83) for black mothers without a breast cancer history and 2.62 (95% CI, 2.01–3.40) for black mothers with a breast cancer history (Table 3).

**Table 3:** PR and 95% CI for preterm birth, low birthweight, and small for gestational age stratified by race/ethnicity

Race/ethnicity	Breast cancer history	Preterm birth^a^ (<37 weeks)		Low birthweight^a^ (<2500 g)		Small for gestational age^a^ (<10th percentile^c^)	
		*n*	PR (95% CI)^b^	*n*	PR (95% CI)^b^	*n*	PR (95% CI)^b^
Black, non-Hispanic	No	75,286	1.00	71,159	1.00	90,006	1.00
	Yes	40	1.31 (1.00–1.72)	41	1.49 (1.14–1.94)	41	1.44 (1.11–1.87)
White, non-Hispanic	No	130,740	1.00	97,273	1.00	123,182	1.00
	Yes	68	2.06 (1.67–2.54)	35	1.53 (1.12–2.08)	27	1.10 (0.77–1.58)
Black, non-Hispanic^d^	No	75,286	1.45 (1.44–1.47)	71,159	1.82 (1.80–1.84)	90,006	1.82 (1.80–1.83)
	Yes	40	1.90 (1.45–2.50)	41	2.71 (2.08–3.54)	41	2.62 (2.01–3.40)

^a^ *P* interaction of race/ethnicity and breast cancer history is 0.01 for preterm birth, 0.9 for low birthweight, and 0.2 for small for gestational age

^b^Adjusted for mother’s age at infant delivery, education, marital status, parity, and smoking

^c^Birthweight of infant is below the tenth percentile for the infant’s gestational age and sex

^d^White women with no breast cancer history serve as the reference group for these comparisons

*CI* confidence interval, *PR* prevalence ratio

In analyses restricted to births to women with a breast cancer history, receipt of chemotherapy was associated with a PR of 1.78 (95% CI, 1.25–2.53) for PTB, 1.68 (95% CI, 0.94–3.03) for LBW, and 1.72 (95% CI, 0.94–3.15) for SGA compared to no chemotherapy. The PR of PTB among births that occurred within 2 years of the mother’s diagnosis date was 2.19 (95% CI, 1.31–3.67) compared to births that occurred 5 years or more after the mother’s diagnosis date. The corresponding PRs were 1.37 (95% CI, 0.58–3.21) for LBW and 0.89 (95% CI, 0.47–1.68) for SGA (Table 4).

**Table 4:** PR and 95% CI for preterm birth, low birthweight, and small for gestational age in women with a breast cancer history

	Preterm birth (<37 weeks)			Low birthweight (<2500 g)			Small for gestational age (<10th percentile^c^)		
	*n*	Age adjusted^a^ PR (95% CI)	Multivariable^b^ PR (95% CI)	*n*	Age adjusted^a^ PR (95% CI)	Multivariable^b^ PR (95% CI)	*n*	Age adjusted^a^ PR (95% CI)	Multivariable^b^ PR (95% CI)
Chemotherapy									
No	38	1.00	1.00	28	1.00	1.00	26	1.00	1.00
Yes	70	1.86 (1.31–2.64)	1.78 (1.25–2.53)	48	1.85 (1.21–2.83)	1.68 (0.94–3.03)	42	1.80 (1.13–2.85)	1.72 (0.94–3.15)
Time between diagnosis and delivery									
<2 years	62	2.24 (1.34–3.74)	2.19 (1.31–3.67)	38	1.47 (0.87–2.51)	1.37 (0.58–3.21)	26	0.88 (0.51–1.53)	0.89 (0.47–1.68)
2–4.9 years	30	1.11 (0.63–1.95)	1.11 (0.63–1.96)	20	0.73 (0.40–1.33)	0.68 (0.28–1.64)	21	0.66 (0.38–1.17)	0.57 (0.30–1.08)
≥5 years	16	1.00	1.00	18	1.00	1.00	21	1.00	1.00
Race/ethnicity									
White, non-Hispanic	68	1.00	1.00	35	1.00	1.00	27	1.00	1.00
Black, non-Hispanic	40	1.00 (0.71–1.42)	0.97 (0.65–1.45)	41	2.02 (1.33–3.05)	1.76 (1.08–2.86)	41	2.60 (1.66–4.08)	2.55 (1.53–4.24)
Parity									
Primiparous (1 birth)	28	1.00	1.00	22	1.00	1.00	21	1.00	1.00
Multiparous (≥2 births)	80	1.27 (0.86–1.86)	1.10 (0.74–1.64)	54	1.09 (0.69–1.72)	0.78 (0.47–1.27)	47	0.94 (0.58–1.51)	0.80 (0.48–1.32)

^a^Adjusted for mother’s age at infant delivery

^b^Adjusted for mother’s age at infant delivery, education, marital status, parity, race/ethnicity, and smoking

^c^Birthweight of infant is below the tenth percentile for the infant’s gestational age and sex

*CI* confidence interval, *PR* prevalence ratio

## Discussion

Three population-based studies of birth outcomes of women diagnosed with breast cancer have been conducted in Western Australia, Sweden, and Denmark [8, 18, 25]. These studies linked cancer and birth data from nationwide registries, yet have discordant results. In Western Australia during 1982–2003, 5% (*n* = 123) of women with a breast cancer history conceived after their diagnosis, 50% (*n* = 62) of them had a birth within 2 years of their diagnosis date, and only two PTB (<36 weeks gestation) were reported [18]. In Sweden during 1973–2002, women with a breast cancer history (when compared to the general population) had greater odds of delivering an early PTB (<32 weeks gestation; adjusted odds ratio (aOR) 3.20, 95% CI, 1.70–6.03) or very LBW (<1500 g; aOR 2.86, 95% CI, 1.41–5.78) infant [8]. This increase was not observed in Denmark during 1973–2002, where the odds of delivering a PTB (<37 weeks gestation; adjusted prevalence odds ratio (aPOR) 1.2, 95% CI, 0.4–3.8) or LBW at term (<2500 g and ≥37 weeks gestation; aPOR 1.3, 95% CI, 0.7–2.2) infant were not significantly different between women with a breast cancer history and the general population [25].

Compared to our study population that includes 512 births to women with a breast cancer history, the Danish study [25] had 695 births and the Swedish study [8] had 331 births to women with a previous breast cancer diagnosis. Our effect estimates were similar to those presented in the Danish study [25] and we used the same cut-off point for PTB, but our effect estimates were statistically significant. The Swedish study [8] used lower cut-off points for PTB and LBW. We re-analyzed our data using the same cut-off points as the Swedish study [8], but our results were not statistically significant. The Danish study [25] conducted stratified analyses by sex of child and type of treatment, but did not see a substantial change in the overall effect estimates. Our analysis reinforces that the racial group is an important consideration for evaluating adverse birth outcomes in the USA.

Examination of the prevalence of adverse birth outcomes within race showed that both black and white mothers with a breast cancer history had a significant increase in risk (31–49% and 10–106%, respectively) of delivering a PTB, LBW, or SGA infant compared to mothers without a breast cancer history within their racial group. Yet, the prevalence ratios of PTB and LBW were higher for white mothers compared to black mothers in these analyses. This may be due, in part, to the cumulative exposure to risk factors for PTB and LBW [42, 43] influencing the health status of black women such that the added health implications of being diagnosed and treated for breast cancer does not have the same level of effect on their reproductive health outcomes as it does for white women. When white mothers in the general population are used as the common referent, white mothers with a breast cancer history have the highest prevalence ratio of PTB, but black mothers with a breast cancer history have the highest prevalence ratio of LBW and SGA. The interaction between breast cancer history and race was significant on the multiplicative scale in the within-race analyses when examining PTB. These results attest to the importance of examining disparities in adverse birth outcomes within racial groups, instead of solely using the white unexposed group as the reference for all comparison groups [44].

Some limitations of our analysis must be considered. Information on breast cancer hormone receptor status, stage, use of endocrine therapy, and chemotherapy agents, dose, and cycle were either not available or frequently missing and therefore not analyzed. Samples sizes were small, with correspondingly limited statistical power in subgroup analyses. Misclassification of the breast cancer history status of the mother was more likely to occur among births that occurred early in our 1990–2009 study period since breast cancer diagnoses prior to 1990 were not included in the data. This misclassification may be largest among women with more than 5 years between their breast cancer diagnosis and infant delivery dates. We were not able to account for multiple births to the same mother. Pregnancies that result from assisted reproductive technology (ART) cannot be identified and tend to have a higher prevalence of multiple births, as well as PTB and LBW deliveries. Only about 1.5% of infants born in the USA general population are conceived via ART [45].

The strengths of our analysis include the use of a population-based dataset to address a 20-year period with approximately two million births overall. With persistent racial disparities in cancer and birth outcomes in the USA, the added exploration of race as a modifier of the association between breast cancer history and adverse birth outcomes is an important contribution. Our analyses that examined receipt of chemotherapy and time between diagnosis date and delivery provide additional insights into factors along the breast cancer trajectory that may contribute to increased risk of adverse birth outcomes among mothers with a breast cancer history. However, we are unable to disentangle the influence of chemotherapy and time since diagnosis since women who gave birth within 2 years of their diagnosis date were slightly more likely to have received chemotherapy (54.9% received chemotherapy and 45.1% did not) than women who gave birth 2 or more years after their diagnosis date (46.1% received chemotherapy and 53.9% did not). In our data, it is also not possible to distinguish pregnancies that co-occur with breast cancer diagnosis or the active treatment period. Future longitudinal studies that follow breast cancer patients from diagnosis to birth will be critical to disentangle potential differences between women who are diagnosed with breast cancer during pregnancy from those who conceive after the active treatment period.

## Conclusions

Women with a premenopausal breast cancer diagnosis may not have started or completed their families at the time of diagnosis. Our findings indicate that a breast cancer history may correspond to 30–67% increases in risk of delivering a PTB, LBW, or SGA infant compared to the general population, with greater increases in risk observed among women who received chemotherapy or gave birth within 2 years of diagnosis.

Breast cancer treatment has long-term implications on the health and quality of life of women. Understanding the effects of breast cancer treatment on future reproductive health outcomes is an important concern for premenopausal women. They may benefit from targeted preconception health services and reproductive health counseling prior to and after cancer treatment. Furthermore, qualitative research may reveal a deeper understanding of the decision-making process of breast cancer survivors regarding their treatment regimen as it relates to their post-treatment childbearing goals.

## Abbreviations

aOR: Adjusted odds ratio
aPOR: Adjusted prevalence odds ratio
ART: Assisted reproductive technology
CI: Confidence interval
LBW: Low birthweight
NAACCR: North American Association of Central Cancer Registries
NC-CCR: North Carolina Central Cancer Registry
PR: Prevalence ratio
PTB: Preterm birth
SD: Standard deviation
SES: Socioeconomic status
SGA: Small for gestational age
USA: United States of America
